# Advances in Patient Classification for Traditional Chinese Medicine: A Machine Learning Perspective

**DOI:** 10.1155/2015/376716

**Published:** 2015-07-12

**Authors:** Changbo Zhao, Guo-Zheng Li, Chengjun Wang, Jinling Niu

**Affiliations:** Department of Control Science and Engineering, Tongji University, Shanghai 201804, China

## Abstract

As a complementary and alternative medicine in medical field, traditional Chinese medicine (TCM) has drawn great attention in the domestic field and overseas. In practice, TCM provides a quite distinct methodology to patient diagnosis and treatment compared to western medicine (WM). Syndrome (ZHENG or pattern) is differentiated by a set of symptoms and signs examined from an individual by four main diagnostic methods: inspection, auscultation and olfaction, interrogation, and palpation which reflects the pathological and physiological changes of disease occurrence and development. Patient classification is to divide patients into several classes based on different criteria. In this paper, from the machine learning perspective, a survey on patient classification issue will be summarized on three major aspects of TCM: sign classification, syndrome differentiation, and disease classification. With the consideration of different diagnostic data analyzed by different computational methods, we present the overview for four subfields of TCM diagnosis, respectively. For each subfield, we design a rectangular reference list with applications in the horizontal direction and machine learning algorithms in the longitudinal direction. According to the current development of objective TCM diagnosis for patient classification, a discussion of the research issues around machine learning techniques with applications to TCM diagnosis is given to facilitate the further research for TCM patient classification.

## 1. Introduction

Traditional Chinese medicine has been used for treatment and prevention of diseases and healthcare for thousands of years in China. To some extent, TCM has also been treated as a popular complementary and alternative medicine in medical field. This is due to the fact that western medicine generally focusing on prescribing medication to deal with the patient's symptoms as effectively as possible. However, TCM theory is based on philosophical frameworks such as the Yin-Yang and five elements theory, the human body meridian systems, and the Zang Fu theory [1, 2], wherein TCM treatments intend to restore the Yin-Yang balance of patient's body and then eliminate the causes of the diseases [3]. To be more detailed, according to the Ying-Yang balance theory, everything consists of five elements: wood, fire, earth, metal, and water. Based on this theory, TCM interprets that the physiology and pathology of human body and the natural circumstance have some relationships which shows the visceral organs having similar properties with the five elements [3]. Thus, diseases would occur if the Ying-Yang balance is disturbed in our body system. Meanwhile, the visceral organs are affected and some clinical manifestations and pathological conditions will appear on some parts of the body or sometimes throughout the whole body.

Then, in order to examine these human pathological conditions in practice, TCM ancient specialists establishe four main TCM diagnostic methods as commonly called inspection (observation), auscultation and olfaction (listening and smelling), interrogation (inquirying or questioning), and palpation (pulse examination). The inspection is to observe all the visible signs and external conditions of the patients which mainly include the vitality, color, appearance, secretions, and excretions. Auscultation and olfaction refer to utilizing the auditory and olfactory sense to gather information about the patient's voice, breathing, coughing, and odor. Interrogation is a way to ask various questions about patients' family history, major complaints, living states, diets, sleeping habits, and such like these physical conditions. Palpation always examines patients' pathological changes of internal organs by doctors' three fingers touching three special positions of radial artery pulse. All these diagnostic methods require considerable skills which would spend many years for beginners to understand the complicated relationships between symptoms and different diseases, even learnt knowledge from distinguished TCM veteran doctors.

Furthermore, apart from the above four diagnostic methods, TCM diagnostics involves another critical component which is known as the differentiation of syndromes (also known as pattern classification or ZHENG differentiation). This concept is established on the four main diagnostic TCM procedures, which is different from the conventional diagnosis approach as western medicine. Figuratively speaking, it is like a bridge to make a comprehensive analysis from four diagnostic methods and then guide the choice of TCM treatment with acupuncture and herbal formula based on TCM diagnostics and treatment theory. In practice, the mentioned four diagnostic methods would derive two critical cues used for syndrome differentiation, which are called symptoms and signs. A symptom is identified as a subjective experience changes in the physiological and psychological functioning, sensations, and cognition of an individual. Oppositely, a sign is referred to as any abnormality indicative of disease which is examined by TCM practitioners [4].

**Figure 1:**
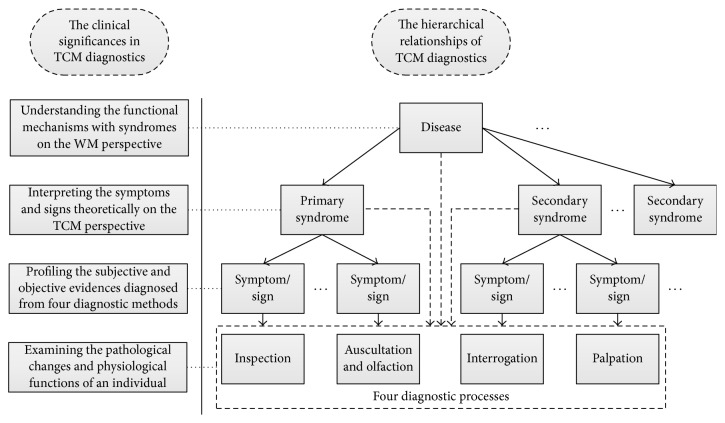
The hierarchical relationships and corresponding clinical significance of TCM diagnostics.

From the perspective of TCM practitioners, both syndromes and diseases should be diagnosed upon the patients' symptoms and signs. This is due to both syndromes and diseases can providing information for making a prescription on the Chinese medical treatment. In particular, a disease indicates pathological changes of patients' body whereas a syndrome reflects the status of a disease at a certain period. Therefore, in general, the relationships among those diagnostic methods and disease can be delineated by a unified framework as shown in Figure 1.

Seen from the hierarchical diagram, it is easy to know that a patient who suffers from a WM disease is a mixture of one primary syndrome with several secondary syndromes on the TCM perspective. And all syndromes are the theoretical profiles of several symptoms/signs (i.e., manifestations) but not only a simple assemblage of symptoms/signs. Moreover, each symptom is a diagnostic conclusion based on the four main diagnostic methods. In particular, the dotted arrow in this diagram is also outlined due to some patient classification studies which focus on syndrome differentiation or disease classification without using all the diagnostic methods. This is reasonable because some syndromes or diseases of TCM reflect apparent and distinct pathological changes on certain aspects, which makes it possible to classify patient only by one or several diagnostic methods, even regardless of the differentiation process of syndrome. In addition, a patient may suffer from several diseases at the same time and one disease can reflect several syndromes, and, furthermore, one syndrome could be transformed during the TCM treatment of the illness, so the syndrome differentiation is also dynamic simultaneously. So in a more comprehensive view, the relationships between TCM diagnostics with diseases should be multidimensional, which has been exhibited in [5].

As one of the main aspects of complementary and alternative medicine throughout the whole medical field, the practice of TCM has been studied extensively for a long time. However, some serious challenges are still hindering the development of TCM diagnosis and treatment. For example, the basic methodology of TCM diagnostic methods is still mainly based on the observation with practitioners' nude eyes or clinical experience knowledge for thousands of years. This is likely to get inconsistent diagnostic results due to the large dependence of practitioners' subjective experiences and personal knowledge. What is more, different patients with the same disease may be subjected to different TCM syndromes; conversely, different diseases may contain the same TCM syndrome. This makes TCM diagnostics difficult to be put into practice, even done by distinguished TCM practitioners. Therefore, it is worthwhile for TCM doctors and scholars to develop an objective and reliable computer-assisted system for clinical diagnosis.

In recent decades, computational methods for TCM have been developed to allow experts to identify and diagnose pathological information and also explore these potential relationships which are unknown between current TCM and western medicine. For these methods, the ultimate purpose is to classify patients with different diseases or syndromes and investigate their potential relationships. Then, on the basis of clinical manifestations, effective TCM treatments could be formulated to restore the balance of patients' body. However, in practice, the successful and appropriate treatments would require accurate syndrome differentiation or ZHENG classification based on the diagnostic symptoms and signs. Thus different TCM treatments could be applied to their corresponding patients classes. Generally, patient classification is critical not only for clinical uniformity and efficacy diagnosed by different TCM experts, but also for the development of TCM objectification which could consummate the understanding of the relationships among different syndrome types, symptoms/signs, and diseases.

The remainder of this paper is organized as follows: Section 2 describes the related works on the survey of TCM modern researches.  Section 3 briefly introduces some classic and advanced machine learning algorithms for researchers' understandings of the characteristics of those techniques. Extensive works on patient classification issue are presented in Section 4, including four main diagnostic approaches, syndrome differentiation from medical records, and some miscellaneous aspects of TCM. Then, we discuss current status and main problems of patient classification with machine learning techniques in Section 5. Finally, Section 6 draws some conclusions for the whole paper.

## 2. Related Works

For the related summarization studies in modern researches of traditional Chinese medicine, a large amount of works has been carried out previously in terms of different aspects of TCM (syndrome differentiation, medical records analysis, four diagnostic methods, treatment, and a mixture of them) [1, 3, 5–15]. In view of practice and applications, machine learning can be derived with two aspects: pattern recognition and data mining. The pattern recognition technology is more commonly used for inspection, auscultation and olfaction, and palpation which attempts to recognize the correct pathological information such as facial complexion, pulse condition, acoustic features, and the chemical components of odor of an individual. Data mining technology, mainly used for text mining and knowledge discovery in machine learning field, focuses on finding out various kinds of hidden knowledge relationships such as symptom and symptom, symptom and syndrome, and syndrome and disease.

From machine learning perspective, several works paid more attentions on surveys about using pattern recognition approaches to perform TCM researches. Lukman et al. [1] presented briefly on TCM elements and reviewed various computational approaches on TCM herbs and formulations, TCM diagnosis, and other biomedical mining systems for TCM. Finally, they concluded that the development of standards for evaluating various computational methods for TCM is urgent and significant. Jokiniemi [3] not only surveyed the TCM herbs, formulations, and diagnosis, but also focused on the expert systems, knowledge acquisition, and sharing systems in TCM. In particular, he also presented some famous corporations deploying technology to help hospitals standardize their patient records in China on the health-care issues, such as IBM, Dell, and Microsoft. This showed that TCM has been paid attentions and has spread worldwide. Jiang et al. [5] focused their survey on syndrome differentiation and pharmacological evaluation of TCM herbal formulary for drug discovery. Several general and impressive frameworks have been summarized based on the current modern researches of TCM. Gu and Chen [6] discussed the relationship between modern bioinformatics and TCM and concluded that the major focuses of current TCM researches are to understand the pathological mechanisms of TCM from the systems biology perspective and facilitate novel drug design based on the analysis of TCM herbal medicine. Ferreira [7] introduced the validation methods of computational models for diagnostic assessment and also analyzed some potential reasons of misdiagnosis results. In another work [10], Ferreira and Lopes reviewed pattern classification researches regarding specific disorders such as genitourinary, cardiovascular, neurologic, surgical, and the like. Lu et al. [8] described historical evolution on the syndrome differentiation, the methodology of syndromes differentiation, and the efficacy of TCM practice with syndromes and diseases. Guo et al. [13] illustrated the methods and status of current objective researches of TCM diagnosis according to the modern TCM diagnostic processes, which contains platform of software and hardware construction, data acquisition, feature extraction from sample data, and syndrome classification. Sá Ferreira [14] evaluated the diagnostic accuracy of Pattern Differentiation Algorithms (PDA) with different combinations of four diagnostic methods, and finally he suggested that both explained and available information should be used as objective criteria for PDA evaluation.

On the other hand, some works provided surveys mainly on data mining methods for TCM. Zhang et al. [9] discussed the data mining methods used on real-world clinical diagnosis and treatment. Lan et al. [11] investigated current data mining applied in TCM clinical diagnosis, syndrome standardization and prediction, and investigation of herbs and formulations. Zhou et al. [12] made a comprehensive overview of the basic TCM concept and a series of theories, related information sources (such as bibliographic literature databases and annotated ancient literature databases) and text mining methods to TCM. They also compared the differences between modern biomedicine and TCM on the viewpoint of methodology. Feng et al. [15] reviewed the knowledge discovery in database for medical formula, herbal medicine, syndrome research, and clinical diagnosis. In addition, they discussed several important issues on where is gold standard, what kind of gold standard is hidden, and how we can mine for the gold standard.

Throughout all the above reviews on the TCM researches, summarizing and inducing the different types of machine learning techniques applied to TCM patient classification on different applications still have not been investigated explicitly. To do this review, valuable information would be beneficial to TCM in several aspects: (1) It could guide TCM practitioner, who expects just to apply machine learning algorithms for specific applications, to choose more general and effective method. (2) TCM researchers could also discover machine learning algorithms which have not yet been studied or used for specific applications. (3) We could understand some domain-related characteristics of TCM clinical data for patient classification problem from a machine learning perspective. In this regard, we aim to review the advanced researches for TCM patient classification according to different machine learning types.

## 3. Preliminary Knowledge for Machine Learning Algorithms

In this section, in order to do preliminary understanding of machine learning algorithms, we will firstly introduce several approaches frequently used for patient classification in TCM. Nevertheless, it is almost impossible to elaborate all machine learning algorithms in this paper, and also not what we should consider to do. So we only describe briefly several classic algorithms and some advanced approaches raised recently. Moreover, those methods are the foundations of most current machine learning algorithms, which can explain the core idea of the majority of other later proposed methods.

*k Nearest Neighbor (k-NN)*. A type of lazy learning, which is regarded as the simplest machine learning algorithm, is implemented by computing the closet training samples in the test samples. It is modeled without any training phase, just only to store the feature (or attributes/variables) vectors and their corresponding labels. In addition, the commonly used distance metric is Euclidean distance and the only parameter is the *k* value.

*Support Vector Machine (SVM) [15]*. A supervised machine learning algorithm constructs a hyperplane or a set of hyperplanes in feature space. It can be often used as classification or regression model. By applying the kernel trick, nonlinear SVM can be derived from the original linear SVM. The effectiveness of SVM depends on the kernel function and several parameters selection like the kernel's parameters and soft margin parameter.

*Linear Discriminant Analysis (LDA)*. It is related to another method called Fisher's linear discriminant which attempts to discover a linear combination of features for classification. It can be used as classification or dimensionality reduction. So it is also similar to principle component analysis (PCA) from the dimensionality reduction perspective.

*Naïve Bayes (NB)*. It is a simplified probabilistic model based on Bayes' theorem but assumes strong independence among all features. The parameter estimation for naive Bayes models always uses maximum likelihood in most cases. Even this algorithm makes a strong independence assumption between the features, it still can achieve excellent classification performance in most practical applications.

*Decision Tree*. A predictive model maps samples' features to class labels which can be built as classification trees or regression trees. It is called tree because the labels are represented as trees' leaves and the conjunctions of features are represented as trees' branches. The tree can be learnt by repeating on different subsets of original feature sets in a recursive manner. Some notable decision-tree algorithms are ID3 (Iterative Dichotomiser 3), C4.5 (successor of ID3), and CART (Classification And Regression Tree). In addition, based on decision tree, some advanced techniques are derived, which is known as ensemble classifier, such as Random Forest.

*Artifical Neural Network (ANN)*. It is inspired by an animal's central nervous system and referred to computational model in machine learning field. It builds a system with a large number of neurons from input to output. The connections of neurons on neighbor layers are modeled by activated functions. Its learning algorithms are common using backpropagation with gradient descent. Common neural networks are Backpropagation Neural Network (BP-NN) and Radial Basis Function Neural Network (RBF-NN).

*Graphical Models*. A probabilistic model which uses graph representation to denote the conditional dependence structure among various features. It can be used to discover the features relationships and analyze the complex distribution of features in feature space. Bayesian networks (BNs), conditional random fields (CRFs), and Hidden Markov Model (HMM) are some famous types of graphical models.

*Multilabel Learning*. It is phrased as a problem of modeling multiple input features mapped into multiple output labels for each sample. So it is opposite to single-label learning and completely different to multiclass learning which outputs different labels for different samples but only one label for each sample. Some famous algorithms are binary relevance (BR), multilabel *k*-NN (ML-*k*NN), Rank SVM (Rank-SVM), and so on.

*Deep Learning*. A recent advanced machine learning technique which contains a set of algorithms, such as Deep Belief Networks (DBNs) and Deep Convolutional Neural Networks (DCNNs). It attempts to build a highly nonlinear transformation to map raw data into high-level abstractions with a large deep network. In particular, it can learn feature representation automatically based on the given raw data. It has achieved great success in recent years on fields of computer vision, automatic speech recognition, and natural language processing.

*Clustering Analysis*. An unsupervised machine learning technique aims to aggregate a set of samples into different groups (clusters). In the same group, all samples are similar but distinct with samples in the other groups based on the specified metric: well known algorithms like *k*-means and fuzzy logic based version known as fuzzy *C*-means (FCM).

## 4. Machine Learning Approaches for TCM Patient Classification

This section gives detailed overview of current researches on TCM patient classification. But firstly, it should be noted that large works focus on symptoms/signs diagnosis using machine learning methods and do not directly carry out the syndrome differentiation or patient classification. So in consideration of the significance of those studies, the survey in this paper for patient classification would be more generalized than previous studies which also contains the symptoms/signs classification based on TCM diagnostics and relationships among symptom, syndrome, and disease. Therefore, patient classification problem will be generalized into three aspects with respect to specific TCM diagnostic data: sign classification (SC), syndrome differentiation (SD), and disease classification (DC) issues. In addition, for the medical records data collected from the four diagnostic methods, most works focus on the SD issue with common machine learning algorithms or their proposed methods. But in fact, there are some researches exploring other aspects from the perspective of data mining technique. Those works attempt to discover some relationships of medical records without conducting syndrome differentiation in TCM: symptom and symptom, symptom and syndrome, and syndrome and disease relationships.

### 4.1. Machine Learning Approaches for Inspection

As mentioned earlier in Section 1, the diagnostic method inspection is to observe some visible signs from patients. Those signs contain various aspects that appeared on the appearance of an individual or excretions from patients. Seen from the current literatures studied on the inspection issue, most works emphasize on the tongue information, lip color, and facial complexion analysis. Classic machine learning algorithms and their modified versions have been proposed to different inspection problems.  Table 1 summaries some of recent studies for TCM inspection, where the number in each table cell indicates the corresponding reference.

Based on this table, it is easy to find that the SVM and *k*-NN methods are more commonly used in the SC issue based on inspection data. Except these algorithms, some other classic algorithms are also utilized to TCM inspection, such as naïve Bayes, decision tree, and neural networks. In particular, several graphical models have been studied and introduced on different TCM diagnosis, such as Bayesian Networks and Hidden Markov Model.

For the specific application on tongue diagnosis, Huang and Li [16] reported that we could use all pixels in tongue surface to analyze tongue color categories, so they proposed a tongue color analysis scheme based on a reduced *k*-nearest neighbor algorithm. Chiu [17] employed feature matching based on *k*-NN metrics to identify the colors of the tongue and the thickness of its coating. Wang et al. [18] showed that regional information is more essential than pixel-wise based tongue color classification. They used Earth Mover's distance algorithm to classify different categories of colors of substances and coatings on tongue. Zhang et al. [19] studied normal health, appendicitis, and pancreatitis diseases classification problem by extracting the chromatic and textural features. Combining feature selection procedure and nearest neighbor classifier, they validate the significant role of tongue inspection for these diseases diagnosis. Kanawong et al. [20] proposed a ZHENG classification system which extracts the coating information as features on modified specular-free tongue images. Different syndrome differentiation issues from gastritis versus healthy volunteers database are built and evaluated upon several classification algorithms including Support Vector Machine, Multilayer Perceptron Networks, and Random Forest. Another similar work [21] compared linear SVM, nonlinear SVM, Radial Basis Functions (RBF) neural network, and *k*-NN to analyze chronic cholecystitis patients and healthy volunteers classification based on hyperspectral medical tongue images. The experimental results manifested nonlinear SVM is more appropriate to their data. Siu et al. [22] derived 24 tongue classes from syndrome perspective and compared five algorithms supported by Weka software: two decision tree-based, two Bayesian-based, and SVM algorithms. As a result, SVM achieved best performance in terms of accuracy and Receiver Operating Characteristics Curve (AUC) evaluation metrics.

All the above studies mainly considered a simple classifier as *k*-NN or a more powerful classifier as SVM. There are also some sparse researches that brought in other machine learning algorithms and models. In the early stages, Jang et al. [23] have introduced neural network to analyze the color of tongue body. Afterwards, neural network has been also employed in [20, 21] as mentioned before. Graphical model, such as Bayesian networks, has been compared to other methods in [22]. Zhang et al. [24] proposed a computer aided tongue diagnosis system (CATDS) which contains acquisition module, image processing module, and diagnosis module. The Bayesian networks classifier is integrated for 9 diseases and healthy subjects diagnosis, similar as [25]. Li et al. [26] focused on the tongue fissure extraction and classification problem, where a Hidden Markov Model is utilized to classify tongue fissures into 12 typical categories. TCM diagnostics is characterized by subjective experience and fuzzy definition in a long period, so fuzzy theory-based algorithms and frameworks may be suitable to explain the potential links of TCM. Hence, the state-of-the-art researches have been studied for TCM in recent decades. Watsuji et al. [27] early built a tongue inspection diagnosis system based on fuzzy rules of TCM items and logic to make different syndromes classification. Pham and Cai [28] introduced some visualization techniques and processes to analyze interactive tongue inspection data. And, also, they discuss how fuzzy set theory can be integrated into visualization techniques to improve perception and understanding of tongue inspection data. More recently, Huang et al. [29] performed tongue shape classification by geometric features. A fuzzy fusion framework was combined with 7 Analytic Hierarchy Process (AHP) modules. This method validated that it can represent the uncertainty and imprecision between quantitative features and tongue shapes. Chiu et al. [30] considered the inspection of the sublingual veins of tongue for blood stasis classification using logistic regression, which is a type of probabilistic statistical classification model. Another tongue color classification model [31] applied spectral angle mapper (SAM) algorithm to discriminate different categories of substances and coatings. This method compares image spectra to known spectra which is applied to hyperspectral tongue images.

Indeed, tongue diagnosis has been studied for a long time. But some recent reports showed that facial diagnosis and lip diagnosis were also considered on the agenda. Preliminary works have been carried out for facial color classification (five morbid color and healthy color) using *k*-NN classifier [32, 33]. They also designed a device for facial image acquisition which aims to overcome the unstable natural light in an open environment. Liu and Guo [34] built an automatic facial color diagnosis system for hepatitis disease; *k*-NN with Euclidean distance is employed as their classifier. Liu et al. [35] compared various supervised learning algorithms on five different facial parts, respectively. The color recognition performance demonstrated that SVM with fusion of five facial parts is more superior than *k*-NN, naïve Bayes, and Adaboost. This conclusion is also similar to another work reported in [36]. The difference is that the facial parts are finer than [35] according to another facial partition TCM theory. Wang et al. [37] investigated normal health and icterohepatitis classification based on facial color by fuzzy clustering and SVM. Zhang et al. [38] not only studied the disease classification based on facial color, but also found that facial gloss information is important to health versus disease classification. They compared SVM with *k*-NN in their experiment, but results showed that *k*-NN is performed slightly better than SVM. Furthermore, Zhao et al. [39] proposed a novel chromatic feature for facial color recognition and gloss analysis based on SVM, which can represent the chromaticity and luminance distribution with facial regional prior knowledge. Zhou et al. [40] focused their work on facial gloss recognition and compared various discriminant analysis methods on different color spaces. The linear discriminant analysis was proved to be more superior competing with others. A more recent study on facial diagnosis has been reported in [41]. This research addressed diabetes mellitus via a more advanced approach than previously, namely, Sparse Representation Classifier (SRC). Based on their method, the average accuracy can reach to 97.54% on the diabetes mellitus versus health classification issue with their facial images database. Moreover, the model based on SRC is more powerful than classic approaches like *k*-NN and SVM. Other than the above researches, Zheng et al. [42] employed SVM to classify different lip color categories with histogram statistical features on various color spaces. Li et al. [43] established an automatic lip color recognition system on a well-designed acquisition device. Different supervised learning algorithms and feature selection algorithms are extensively compared on their database. The conclusion is that SVM with recursive feature elimination for feature selection is the best model for lip color classification.

Throughout all the mentioned works on the inspection issue, we note that a large amount of works focuses on the tongue diagnosis. Meanwhile, the facial diagnosis and lip diagnosis have been reported and studied recently. The popular machine learning algorithms, such as *k*-NN and SVM, are still the first choice seen from the current literatures. Besides, the nonlinear model may be more appropriate to inspection data in most cases according to the above reviewed literatures, which indicates that the spatial structure of inspection data is nonlinear distribution. Furthermore, more advanced machine learning algorithm is also necessary to be studied for TCM, which may improve the previous system performances as shown in [41].

### 4.2. Machine Learning Approaches for Auscultation and Olfaction

In this section, we review another important diagnostic method which contains two subjects: auscultation and olfaction. Auscultation is to examine the voice changes through physician's auditory sense, and olfaction is to check the odor changes through physician's smell sense. Their theoretical foundation is that TCM believes the speech sound and body odor produced by patients can reflect the physiological and psychological conditions of Zang Fu organs. So auscultation and olfaction have caused great attention during a long period in TCM field. However, related works on objective auscultation and olfaction are still sparse and rarely studied. This may be due to the complex acoustic characteristics of sound including massive noises and similar acoustical signals in the nature, and diverse chemical compositions of exhaled breath containing thousands of volatile organic compounds (VOCs). All these factors hinder the development of the research of objective auscultation and olfaction in TCM. To our best endeavors, a minority of preliminary researches have been studied on this issue. Even so, we list related works in Table 2. Besides, detailed descriptions are presented in objective auscultation and olfaction perspective, respectively. In addition, although some works did not even learn a machine learning model for objective auscultation-olfaction diagnosis, we will still review them briefly because their fundamental works would be a valuable reference for further researches in the future.

For the objective auscultation analysis, Wang et al. [44] have reviewed some digital techniques in recent years. But almost all of the related works on auscultation diagnosis are reported before the 21st century, where the researches are preliminary and immature. More recently, some auscultation signals analysis works in TCM have been reported. Yan et al. [45] introduced wavelet packet energy entropy for decomposing auscultation signals to split more elaborate frequency bands. Then SVM is utilized to classify patients into their respective categories: health, Qi-vacuity, and Yin-vacuity. In another work, Yan et al. [46] considered the nonstationarity information of the auscultation signals to automatically recognize healthy individuals from the one with Qi-deficiency or Yin-deficiency. By means of SVM and a feature selection method conditional mutual information maximization criterion, they obtained 80% classification accuracy based on their experimental results. Furthermore, one improved feature extraction work on auscultation signals has been studied in [47]. The combination of wavelet packet transform and two different entropy methods (sample entropy and approximate entropy) was computed to quantize all audio signals. And, likewise, they performed classification with SVM on the same issue. Finally, the recognition accuracy was improved and approaching 90% with approximate entropy scheme, and even higher than 90% with sample entropy compared to the previous works.

Different from the above works, Chiu et al. [48] proposed four novel acoustic parameters to form signal features: two temporal parameters (the average number of zero-crossings, the variations in local peaks and valleys) and two other parameters (the variations in first and second formant frequencies, and the spectral energy ratio). The classification purpose is tha same as the above works, but another machine learning algorithm logistic regression was introduced to build the recognition model. Considering the speech characteristics related to production irregularities, Chiu et al. [49] also utilized the fractal dimension parameter. Experimental results with logistic regression showed the classification rate was better than their previous work. Yan et al. [50] found that most existing approaches were limited to analyze a single vowel, so they introduced multi-instance multilabel learning framework in order to make a comprehensive analysis with five vowels. The experiment results reveal that this method is effective in identifying the health, Qi-deficiency, and Yin-deficiency auscultation data of TCM. The remainder works we retrieved and did not build a diagnosis model for auscultation diagnosis, such that Yan et al. [51] utilized independent component analysis to noise reduction. Yan et al. [52] introduced the delay vector variance method for detecting the nonlinearity of the time series. Two novel parameters, energy ratio and max power ratio, are proposed to study the objective auscultation in [53].

For the objective olfaction analysis, there are almost no recent works with respect to TCM perspective. Most researches for exhaled breath analysis are carried out for disease diagnosis from the western medicine perspective. Based on this situation, we still review several recent works as a reference for TCM olfaction diagnosis. In disease diagnosis, most works focus on the diabetes identification from healthy volunteers. Ping et al. [54] introduced a nonsupervised fuzzy clustering algorithm for diabetes based on electronic nose in early time. Yu et al. [55] developed a portable gas analysis system by using conducting polymer sensor array. The diabetes patients were discriminated from normal persons by the principle component analysis (PCA) with *k* nearest neighbor classifier. Another breath analysis system [56, 57] was established for two purposes: diabetes diagnosis and blood glucose levels prediction. Their diagnosis models were built by support vector classifier and support vector regression, respectively. Guo et al. [58] applied a novel regression algorithm named support vector ordinal regression to perform classification with four ordinal categories marked with well controlled, somewhat controlled, poorly controlled, and not controlled.

Other researches paid attentions to diverse diseases diagnosis with various machine learning algorithms. Dragonieri et al. [59] used electronic nose to collect exhaled breath for different levels of asthma patients diagnosis and was classified by linear discriminant analysis. An ensembling decision method was established based on soft-margin SVM with Gaussian kernel in [60], and then this model was applied to ten different bacteria cultures captured from electronic nose. Saraoglu et al. [61] used RBF neural network for HbA1c parameter predictions and glucose parameter predictions based on quartz crystal microbalance sensor. Patients with lung cancer could have accelerated catabolism of volatile organic compounds by the induction of high-risk cytochrome p450 genotypes. Therefore, based on this fact, Phillips et al. [62, 63] constructed a predictive model and a fuzzy logic model, respectively, for lung cancer detection and prediction, both with volatile markers. A breath analysis system [64] was built for diabetes, renal disease, and airway inflammation classification based on a simple classifier, known as *k* nearest neighbor. Lin et al. [65] concerned the uremia diagnosis by electronic nose. They collected exhaled breath from normal subjects, uremic, renal insufficiency, and chronic renal failure patients. Final signals analysis was carried out by a standard technique referred to as discriminant analysis.

As can be seen from Table 2 and reviewed researches on the objective auscultation and olfaction diagnosis, the works focused on the TCM are not so many and preliminary. On the contrary, quite a few reports have been studied on the olfaction but were carried out from the disease diagnosis of western medicine perspective. Several common machine learning algorithms are also taken into account on the various disease classification issues, such as SVM, *k*-NN, and ANN. Meanwhile, we find that there are sparse works concerning the issue of sign classification by using auscultation or olfaction data. Although the objective auscultation and olfaction diagnosis from TCM perspective has been seldom put into practice, the above studies could be a knowledge worth learning and exploring.

### 4.3. Machine Learning Approaches for Palpation

Palpation diagnosis (or called pulse diagnosis), another important diagnostic method in TCM, is also a noninvasive and effective way to examine the location and extent of an individual's health conditions. The traditional examined methods in Chinese medicine are feeling three measurement positions on the radial artery defined in TCM theory, where they are known as Cun, Guan, and Chi in Chinese. Several different palpation data acquisition systems have been developed using various signal sensors [66–70]. According to the retrieved papers, we produce the rectangular reference list for the recent machine learning approaches for palpation, as listed in Table 3.

Seen from the table, it is obvious that the sign classification and disease classification based on palpation are the main focus in TCM. However, to our best knowledge, syndrome differentiation by palpation data using computational methods has not been studied yet. This is not as the inspection data for syndrome differentiation which has been investigated as shown previously. On the other hand, commonly used algorithms like SVM and *k*-NN are also the first choice to analyze pulse signals as well as the inspection data. It is worth noting that some advanced machine learning algorithms that caused a great attention recently are also employed to solve pulse waveform classification or disease classification. Therefore, we will overview most works in this part from different aspects including the same issue solved by different machine learning algorithms and different issues solved by same machine learning algorithms.

As for the pulse waveform type classification, Zhang et al. [71] proposed two novel *k* nearest neighbor-based approaches using edit distance with real penalty. Then, those methods were applied to recognize five pulse patterns, including moderate, smooth, taut, hollow, and unsmooth. Compared with other recent literatures, the proposed methods performed better with the accuracy criterion. A decision tree method is introduced in [72] to pulse strength types classification from four hundred pulse signal samples. Due to the imbalanced classes of this dataset, they undersampled the majority class and built a balanced pulse subset. Experimental results on normal strength pulse (NS-pulse) versus feeble pulse (F-pulse) and overall samples classification were both obtained over 90%. Jia et al. [73] recognize five distinct pulse patterns from 2470 pulse waveforms. They provided a novel elastic metric for SVM to perform pulse waveforms classification. Experiments were carried out on two aspects: metrics comparisons and classifiers comparisons. All results demonstrated the proposed metric is appropriate to represent the pulse waveforms data. Xu et al. [74] and Wang et al. [75] all proposed the combination of fuzzy theory and neural networks to address pulse patterns recognition issue. Compared with back propagation network, the fuzzy logic was proved more useful to pulse analysis in TCM. Ling et al. compared various neural networks with the improved Echo State Network (ESN), which is based on the chaos theory, validated the effectiveness, and superiority of ESN neural network. Ma et al. [76] proposed an improved two-step classification method to classify seven common pulse patterns. They first compared eight discriminant functions including SVM, *k*-NN, and decision tree. Then a coarse-to-fine hierarchy classification model is built with optimal classifier in corresponding domains. Final results showed that the improved method could obtain good performance on the overall classification accuracy.

Bayesian networks, a probabilistic graphical model, could build the mapping relationships between pulse types and pulse signals. Wang and Zhang [77] presented a model using Bayesian networks to identify depth, frequency, rhythm, strength, and shape of human pulse signals, respectively. Thus, five graphical structures are established to realize the automatic identification of different parameters of pulse signals. The confusion matrix and accuracy criteria demonstrated the powerful modeling ability of Bayesian networks. Wang and Cheng [78] claimed that the pulse types can be classified into eleven types according to seven factors. They considered that the architecture of the pulse diagnosis system based on BNs should consist of three steps: discovering dependency relationship module, parameter learning, and reasoning module. So modified Greedy Bayesian Pattern Search algorithm (GBPS^∗^) was used for the first two steps; Clique Tree Propagation algorithm (CTP) was implemented for the reasoning module. In addition, they also employed Markov blanket to perform causal inference. All predictive results validated the effectiveness for pulse diagnosis of the proposed system.

For the other purpose of classifying diseases from healthy persons or between the diseases, Yang et al. [79] utilized the independent component analysis to extract pulse feature for cholecystitis and gastritis diagnosis. Modeling with *k*-NN, the best classification recognition rate is obtained by the proposed feature extraction method competing with linear discriminant analysis and principle component analysis. Liu et al. [80] adopted a recent time series matching method and time warp edit distance, to diagnose four disease patients from healthy person with nearest neighbor classifier. Experimental results manifested that the introduced method is superior to other time series matching approaches. A mobile healthcare system was developed in [81] for cirrhosis diagnosis including signal denoising and baseline wander removal for wrist-pulse preprocessing; the proposed feature extraction algorithm is called binning method and *k* nearest neighbor classifier. Sun et al. [82] focused on the feature extraction for pulse analysis using kernel PCA and five diseases classification using *k*-NN.

Apart from *k*-NN for disease classification, a large amount of works prefers to adopt SVM to model their disease diagnosis system. Jiang et al. [83] investigated six classifiers to distinguish patients with gastritis and cholecystitis from the healthy persons. All classifiers include Fisher Linear Discriminant (FLD), Quadric-Programming Fisher linear Discriminant (FLD-QP), Batch-Mode Perceptron (Perc), Kozinec's perceptron (Kozi), *k*-NN, and L2-soft SVM. In three classification tasks, the performances of L2-soft SVM were better than others. Wang et al. [84] extracted lots of different features including shape, width, energy, frequency, wavelet coefficient, and PCA. The *k*-NN and RBF-SVM are employed to separate the health persons from diabetics. Final classification accuracy exhibited the fusion of all the above features and RBF-SVM achieved highest performance on this issue. Similarly, Chen et al. [85] proposed a modified autoregressive model to extract pulse signal feature. By means of SVM, they were able to distinguish healthy persons from acute appendicitis patients with over 82% accuracy and even higher accuracy for the other diseases. Jia et al. [86] fused three types of features on the decision level for disease versus health classification issue, referred to as spatial features, wavelet energy, and similarity features. The Bayes sum rule with SVM achieved best performance compared to other fusing rules in their experiments. Jiang [87] attempted to extract other features called five clinical Doppler parameters (DP), Wavelet Energies (WE), Wavelet Packet Energies (WPE), and Piecewise Axially Integrated Bispectra (PAIB). They input all features into L2-SVM for Doppler blood flow signal analysis. Actually, other works also focus on the feature extraction approaches in order to represent the pulse signals as accurately as possible, such as Hilbert-Huang transform (HHT) [88], Auto Regressive Prediction Error (ARPE) [89], and Wavelet (packet) Transforms (WT) [90] for Doppler ultrasound blood flow signal, multiscale sample entropy [91], and HTT combined with spatial features [92] for wrist pulse blood flow signal. Zhang et al. [93] have designed and implemented a Chinese wrist-pulse retrieval system using pressure sensors, called EasiCPRS. They demonstrated the system architecture and evaluated the pulse diagnosis performance with linear SVM to classify six categories: healthy, subhealthy, hypertension, coronary heart disease, pregnancy, and liver cirrhosis.

In addition, as for the other machine learning algorithms, Chen et al. [94] utilized an unsupervised learning method, fuzzy *C*-means, to directly aggregate all pulse data into three classes: health, pancreatitis, and duodenal bulb ulcer. By using the modified Gaussian model to feature extraction, their approaches provided a better classification performance than the wavelet transform and the autoregressive methods. Liu et al. [95] introduced Multiple Kernel Learning (MKL) algorithm to combine multiple features to perform pulse symptom classification and disease classification. Through different experimental results, the MKL framework achieved the best overall results competing with SVM and *k*-NN. More recently, Deep Convolutional Neural Networks (DCNN) were introduced into wrist pulse signals analysis [96], which is a type of representative learning in machine learning field. Based on this method, accuracy could achieve 72.31% on distinguishing health versus subhealth issue, and 96.33% on arteriosclerosis versus nonarteriosclerosis issue.

In summarization, objective palpation diagnosis has been studied extensively on the sign classification and disease classification. However, the research on the syndrome differentiation issue is seldom retrieved according to our current work. Besides, we also found that most works are concerned with the feature representation of pulse signals and rarely emphasized on the learning model construction on the basis of specific characteristics of pulse data.

### 4.4. Machine Learning Approaches for Interrogation and Medical Records

In this section, we will review the recent advances on interrogation and medical records analysis. Interrogation diagnosis (or called inquiry diagnosis) is to directly ask questions on various physiological and psychological feelings of patients. TCM experts who collect all these information could understand the medical history and present the disease, so as to provide evidences for syndrome differentiation. Medical records rely on the gathering of clinical information through four diagnostic methods. Both symptoms and signs of a patient would be examined with computational methods and recorded in the clinical database.

Sincerely, interrogation data are always one part of the medical records and known as one of the important diagnostic method in TCM diagnostics. But based on the retrieval of related literatures, we notice that few works are reported and investigated objective interrogation analysis for the issues of syndrome differentiation or disease classification purely. In most cases, the interrogation data are always combined with other diagnostic data to study these issues. Hence, we put interrogation and medical records reviews together. But certainly, a few of researches on the interrogation analysis regardless of other diagnostic data would be separately reviewed in the following.

Besides, we also notice that a majority of medical records data analysis are carried out for syndrome differentiation issue, which is essential purpose studied in objective TCM research. Meanwhile, sparse researches are reported to study some relationships based on medical records without conducting syndrome differentiation in TCM: symptom and symptom, symptom and syndrome, and syndrome and disease relationships. These works are also significant and we will present them separately. In general, we will summarize the retrieved articles to formulate a reference with respect to algorithms and their applications, as shown in Table 4. This table is slightly different from the previous table we made due to the distinct applications of medical records.

For the objective interrogation diagnosis, the data collection is generally carried out by inquiry diagnosis scale or questionnaire designed by TCM experts [97]. Li et al. [98] investigated the symptom-syndrome interactions for inquiry diagnosis of coronary heart disease (CHD). On the one side, they first built a large symptom-symptom interaction (SSI) network which reveals their potential connections. Then, on the other hand, the relationship among syndromes was also calculated by means of relative associated density (RAD). RAD was also utilized to show the connections between syndromes and symptom. Based on the above quantitative analysis, in the final stage, RAD was used for symptom selection and both SVM and *k*-NN were employed to predict syndrome from symptoms. Liu et al. [99] proposed a novel feature selection approach combined association analysis and information gain (IG) to perform relevant symptom selection for syndrome differentiation of CHD. Based on *k*-NN classifier, the proposed method achieved the best performance compared to document frequency (DF), IG, and mutual information (MI) feature selection methods.

For CHD syndrome differentiation, considering the multiple syndromes for each patient, Liu et al. [100] introduced multilabel learning algorithms to address this issue. Several multilabel learning algorithms are implemented in CHD dataset including multilabel learning *k*-Nearest Neighbor (ML-*k*NN), Support Vector Machine for Ranking (RankSVM), and backpropagation multilabel learning (BPMLL). Experimental results showed that multilabel learning is superior than single-label learning, and ML-*k*NN achieved better performances than RankSVM and BPMLL. Shao et al. [101] also used multilabel learning algorithms for CHD but focused on the symptom selection algorithms research. They proposed a multilabel feature selection algorithm called hybrid optimization based multilabel feature selection (HOML). A more advanced technique has been introduced for syndrome diagnosis of chronic gastritis [102], which is popular recently in machine learning area. The deep learning model Deep Belief Network (DBN) was combined with binary relevance method, a multilabel learning algorithm, to recognize six common syndromes. Overall performances on various multilabel criteria testified the powerful learning ability of the proposed method for inquiry diagnosis.

As for the medical records data analysis, most works design their systems and approaches to address syndrome differentiation problem. Yang et al. [103] developed the information management system of TCM syndrome which incorporated prior knowledge of TCM syndrome information to SVM and built a P-SVM model to classify TCM literatures as different syndromes. The accuracy rate is 95% on the sample set of 2000 records. Xia et al. [104] compared SVM with stepwise regression and neural network; experimental results indicated RBF kernel function with SVM was the best classifier to identify ten syndromes. Some other works [105, 106] studied the dimensionality reduction algorithms with SVM to improve the syndrome differentiation performance. Wang et al. [107] considered analyzing the raw free-text clinical records, which was very different from lots of well-structured datasets that were manually collected, structured, and normalized by TCM experts. Direct usage of existing diagnostic frameworks was impossible to clinical records. Therefore, they developed a novel automatic diagnosis framework to deal with this unstructured dataset. The architecture of diagnostic system is composed of four components: the TCM symptom names recognition, normalization of symptom names, feature selection, and training/diagnosing modules. First two modules transformed the raw data into well-structured data and latter two were utilized to build an effective syndrome differentiation model. Two classifiers (NB and SVM) are employed to evaluate the effectiveness and feasibility of the proposed system. Wang et al. [108] compared several classifiers to predict syndrome for liver cirrhosis from three perspectives: TCM, WM, and their combined views. The classifiers are logistic regression (LR), BNs, NB, RBF-NN, C4.5, and SVM. Final classification accuracy indicated that the combined features of TCM and WM can achieve the highest performance.

In recent years, several multilabel learning algorithms are used for syndrome differentiation for medical records analysis. Li et al. [109] realized the multisource data of medical records of TCM including data sources from facial and tongue diagnosis, palpation diagnosis, inquiry diagnosis, and other information. Hence, feature level information fusion scheme was proposed based on multilabel learning algorithms. Experimental results validated that it was critical to process different source data separately for ZHENG classification. The multilabel algorithm relevant feature for each label (REAL) was introduced to study the syndrome classification and identification for cardiovascular disease [110]. Wang et al. [111] used a different multilabel learning algorithm to diagnose chronic fatigue via Conformal Predictor with Random Forest (CP-RF). Extensive experiments validated the CP-RF outperformed ML-*k*NN and other CP models with NB and *k*-NN.

Similarly, H. Wang and J. Wang [112] proposed a novel symptom selection algorithm for patients records syndrome differentiation and key element prediction. They utilized GBPS^∗^ algorithms to learn BNs and built a key element-blood stasis Markov blanket. The predictive accuracy rate validated the effectiveness of the proposed quantitative method. A self-learning diagnosis system was developed in [113], which was characterized by data-driven nature and learning knowledge. Improved hybrid BNs were proposed and combined with some knowledge discovery methods to identify syndromes of five representative patient cases. Based on the data-driven nature and knowledge discovery property, their system performed well in TCM diagnosis compared with other existing TCM rule-based systems. The BNs was combined with classic feature selection algorithm in [114], which was applied to three syndromes identification on Chronis Hepatitis B in TCM.

Expect for the above machine learning algorithms, Wang et al. [115] introduced decision tree to liver and kidney yin deficiency syndrome, damp heat smoldering syndrome, and stasis and heat smoldering syndrome. Shi and Zhou [116] proposed a modified BP neural network to syndrome differentiation, but only simulation experiment was reported. An attribute hierarchy model was employed to build the hierarchical diagnosis model for posthepatic cirrhosis data with three levels: patient level, symptom level, and diagnosis level [117]. An interval-valued cloud model, considered as an improvement of fuzzy theory, was adopted to diagnose eight subhealth syndromes in [118]. And the experimental results showed that this model achieved higher performances competing with their previous model trained by BP neural network. Wang et al. [119] designed a hierarchical model of syndrome differentiation with hypergraph in cluster and attributes combination in association procedure. Zhang et al. [120] used latent tree model for analysis of kidney deficiency data. Manifold ranking was proposed to explore the syndrome differentiation for viral hepatitis and compared with PCA, NB, association rules, and *k*-means [121].

Syndrome differentiation may be the core purpose for medical records in TCM. However, some works were also reported without syndrome identification experiment. Their objectives are to mainly explore the relationships between symptoms and symptoms, symptoms and syndromes, or syndrome and disease. Wang et al. [122] built two relationships for coronary heart disease by using SVM: relation between symptoms and syndromes and relation between syndromes and signs from tongue and pulse data. Works in [123, 124] both constructed Bayesian networks to study the associations between symptoms and symptoms, one for kidney disease and another for apoplexy and Chinese medicinal herbs. Cluster analysis was used to study the symptoms related to syndromes for unstable angina [125]. Zhang et al. [126] utilized latent tree models to validate the TCM theory by constructing symptoms and syndromes latent structure. Rough set theory was introduced in [127] to obtain the relationships between syndromes and syndrome elements. Wu et al. [128] used bootstrapping and term cooccurrence to produce the associations between genes and kidney Yang Xu symptom complex.

According to the above reviews and reference listed in Table 4 on interrogation and medical records, it is apparent that syndrome differentiation is what those data usually are used to study. The machine learning techniques have been extensively explored to address this issue in TCM. Even some sophisticated and progressive model learning approaches are investigated for syndrome differentiation, such as multilabel learning and deep learning. Wherein, associations among symptoms, syndromes, and diseases are also analyzed to facilitate the syndrome classification of symptoms and signs collected from different diseases. Furthermore, some TCM experts have proceeded to discover the syndromes which may be related to some genes and proteins in WM perspective.

### 4.5. Machine Learning Approaches for Miscellaneous Applications

Apart from the four diagnostic data and medical records data used for patient classification, some other works are indirectly related to TCM diagnosis. Some literatures explored the relationships between syndromes and herbs or formulas (prescriptions), others expected to discover useful and latent knowledge from text, clinical data, or TCM experts. Considering the significance of these researches in TCM, we will review several works in this section briefly.

Zhang et al. [129] applied a hierarchical symptom-herb topic (HSHT) model to analyze clinical diabetic data. They constructed a hierarchical symptom-herb topic model to describe the latent structures with both symptoms and their corresponding herbs. As a result, this model could provide a computer-aided patient treatment system by recommending some herbs for TCM practitioners. Liang et al. [130] introduced a decision tree with kernel mapping method to discover the underlying relationship between clinical outcomes and symptom types on acupuncture for neck pain. Three questionnaires were applied as measured outcomes for evaluating the acupuncture effect, and nondominated sort algorithm is adopted to keep consistent ranking for the effect with these measurements. According to the experimental results for the classification task of therapy records, we found that this model provided an effective way for outcomes of acupuncture prediction. A knowledge discovery system named KISTCM [131] attempted to discover several relationships for TCM treatment. A novel algorithm called Medicine Dependency Relationship Evaluation (MDRE) was proposed to mine the dependency associations among medicines. Meanwhile, another algorithm GEP which combines genetic algorithms (GA) and genetic programming (GP), was introduced to explore formula-syndrome relationships for TCM treatment. Some major experiments have proved that the KISTCM system was useful and promising for the development of TCM knowledge discovery. Zhang et al. [132] proposed a symptom-herb-diagnosis topic (SHDT) model to automatically construct the potential associations among symptoms, herbs, and diagnosis based on a large-scale clinical diabetes data. Wang et al. [133] carried out a preliminary research on symptom name recognition by using conditional random fields. Final experimental recognition *F*-measure was approaching 63% with recognition rate 93.403%.

Regardless of symptoms and syndromes, some persons focus other aspects for patient treatment research. Fang et al. [134] developed a highly complicated database called TCMGeneDIT to discover the relationships among medicines, genes, diseases, TCM effects, and TCM ingredients from a large amount of biomedical literatures. A herb-herb network was built in [135] to find the core effective formula by using genetic algorithm from a lung cancer dataset. All the results manifested the proposed network that is effective and agreed with the TCM theory. Besides, Tang et al. [136] expected to mine TCM masters knowledge for understanding the TCM diagnosis and treatment. In order to achieve this purpose, they proposed a preliminary framework of TCM master miner which integrated with correspondence analysis, graph theory, and complex networks analysis. Building a comprehensive TCM expert systems is strongly necessary and has been investigated for a long time, but lots of systems are not overall and intelligent as we expected. Huang and Chen [137] proposed a relatively unifying framework for intelligent disease diagnosis system named CMDS (Chinese Medical Diagnostic System for digestive system). This system has integrated various aspects of TCM such as symptoms, identification, treatments, prevention methods, and prescription. They also tested cases to compare the proposed system with diagnosticians. Experimental results indicated that they obtained almost identical answers from CMDS and the diagnosticians.

## 5. Discussions

In order to achieve effective TCM treatment for patient, patient classification is critical issue which has been studied for recent decades. Three subissues (sign classification, syndrome differentiation, and disease classification) are extensively researched according to different TCM source data. We consider the four main diagnostic methods and their fusion medical records data. After the survey from a machine learning perspective, we find out several situations and issues of current objective researches on patient classification for TCM, which are summarized as follows:

1. For the four diagnostic methods, a large amount of works focuses on the inspection and palpation by using various machine learning algorithms. In order to make sign classification, syndromes differentiation based on specific disease and disease classification, inspection concerns the tongue, and face and lip diagnosis, palpation considers using the positions of Cun, Guan, and Chi of radial artery pulse, respectively or entirely. On the contrary, researches on auscultation and olfaction and interrogation are sparse. There are two possible reasons in our perspective. One reason may be the diverse chemical compositions of exhaled breath containing thousands of volatile organic compounds (VOCs) for olfaction, massive noises, or similar acoustical signals for auscultation and the difficulty of standardized inquiry scale for interrogation. Another reason we consider may be the limited disease entities related to auscultation and olfaction. Hence, it may be more apparent and more easier to study objective inspection and palpation in TCM.
2. For medical records analysis, the application for syndrome differentiation is the main purpose in TCM. Moreover, exploring the associations among symptoms, syndromes, and diseases has been also studied for discovering the potential knowledge of medical records. Meanwhile, some recent works also manifest the genes and proteins in WM perspective that are related to syndromes in TCM. This would help us with deeper understandings of the TCM theory.
3. For the other applications, their works are not directly related to patient classification problem. But based on the medical records and other clinical data, they always build some association models among syndromes, herbs, formulas, medicines, genes, diseases, TCM effects, TCM ingredients, and the like. All these researches are critical for TCM diagnosis and treatment after patient classification, so we also should refer to these works for facilitating the unifying system development of patient classification and treatment.
4. From the machine learning perspective, a variety of learning algorithms are introduced to process those TCM data. Some works also proposed appropriate algorithms according to the special characteristics of TCM data. More recently, several advanced machine learning techniques are applied to solve TCM patient classification, such as multilabel learning and deep learning.
5. According to the reviewed works, most of them do not study the machine learning for model construction. For instance, objective inspection and palpation analysis researches put more emphasis on feature representation. Medical records data analysis cares for how to select the optimal symptom subset for syndrome differentiation. But for the structure and distribution characteristics analysis of TCM data, minor works have engaged in these issues. Whereas the more detailed and deeper the researches on structure learning are, the more prominent the diagnosis system would be developed.
6. Based on the current researches, there are no published TCM database reported to provide a benchmark for different diagnosis system evaluations. All works are carried out on their own database built by their respective data acquisition system. This is not beneficial to establish patient classification gold standards which is urgent to be studied.
7. In addition, even a large amount of TCM diagnosis system is developed by computational methods. Meanwhile, most of them claimed that their methods or systems could analyze TCM data from a quantitative perspective. Actually, none of them could quantize their diagnostic data with meaningful implications corresponding to TCM theory, as the clinical indicators from a western medicine perspective. If this situation could not be improved, the establishment of diagnosis standards for TCM may be very difficult. Moreover, it may also hinder the development of objective TCM diagnosis research.

## 6. Conclusions

In this paper, we survey various works related to patient classification issue in traditional Chinese medicine from a machine learning perspective. We first elaborate the basic diagnosis methods and concepts between traditional Chinese medicine and western medicine. Then we illustrate the hierarchical relationships and corresponding clinical significance of TCM diagnostics for better understandings for TCM diagnosis. Afterwards, several common and advanced machine learning techniques are briefly introduced for understandings of their preliminary knowledge. Then, we discuss that the patient classification issue could be divided into three main aspects: sign classification, syndrome differentiation, and disease classification. According to these subissues, we review related works on five different TCM diagnostic data directly related to patient classification from machine learning perspective: inspection, auscultation and olfaction, palpation, interrogation and medical records, and some miscellaneous applications which are indirectly to patient classification.

Finally, based on the above overviews, some current research highlights and existing issues are discussed for further improvement of TCM diagnosis. Actually, due to a large amount of works on patient classification, the current survey in this paper may been not completed and need to be improved further. Nevertheless, it is enough to reflect the current advances in patient classification for TCM. For the comprehensive analysis of current TCM diagnosis for patient classification, we would complement our reviews and complete the current overview tables in the future work.

## Figures and Tables

**Table 1:** Overview of machine learning algorithms for patient classification using inspection (SC_Inspection: sign classification based on inspection; SD_Inspection: syndrome differentiation based on inspection; DC_Inspection: disease classification based on inspection).

Algorithms	Applications		
	SC_Inspection	SD_Inspection	DC_Inspection
*K*-nearest neighbor	[16–18, 29, 32, 33, 35, 43]		[19, 21, 34, 38, 138]
Naïve Bayes	[35, 43]	[22]	
Decision tree		[20, 22]	
Support vector machine	[35, 36, 39, 42, 43]	[20, 22]	[21, 37, 38]
Neural network	[23]	[20]	[21]
Graphical models	[26]	[22]	[24, 25]
Miscellaneous	[29–31, 35, 40]	[27]	[28, 41]

**Table 2:** Overview of machine learning algorithms for patient classification using auscultation or olfaction (SC_Aus-Olf: sign classification based on auscultation or olfaction; SD_Aus-Olf: syndrome differentiation based on auscultation or olfaction; DC_Aus-Olf: disease classification based on auscultation or olfaction).

Algorithms	Applications		
	SC_Aus-Olf	SD_Aus-Olf	DC_Aus-Olf
*K*-nearest neighbor			[55, 64]
Naïve Bayes			
Decision tree			
Support vector machine		[45–47]	[56–58, 60]
Neural network	[61]		
Graphical models			
Miscellaneous		[48–50]	[54, 59, 62, 63, 65]

**Table 3:** Overview of machine learning algorithms for patient classification using palpation (SC_Palpation: sign classification based on palpation; SD_Palpation: syndrome differentiation based on palpation; DC_Palpation: disease Classification based on palpation).

Algorithms	Applications		
	SC_Palpation	SD_Palpation	DC_Palpation
*K*-nearest neighbor	[71, 76]		[79–83]
Naïve Bayes			
Decision tree	[72, 76]		
Support vector machine	[73, 76]		[83–93]
Neural network	[74, 75, 139]		[84, 140]
Graphical models	[77, 78]		
Miscellaneous	[76, 95]		[79, 83, 94–96]

**Table 4:** Overview of machine learning algorithms for patient classification using interrogation or medical records (SD_Int-MRs: syndrome differentiation based on interrogation or medical records; AA_Int-MRs: association analysis among symptoms, syndromes, and diseases based on interrogation or medical records).

Algorithms	Applications	
	SD_Int-MRs	AA_Int-MRs
*K*-nearest neighbor	[98, 99]	
Naïve Bayes	[107, 108, 121]	
Decision tree	[108, 115]	
Support vector machine	[98, 103–108]	[122]
Neural network	[104, 108, 116]	
Graphical models	[108, 112–114]	[123, 124]
Multilabel learning	[100, 101, 109–111]	
Miscellaneous	[102, 104, 108, 117–121]	[98, 125–128]
